# Prognostic Assessment of Gastropancreatic Neuroendocrine Neoplasm: Prospects and Limits of Radiomics

**DOI:** 10.3390/diagnostics13182877

**Published:** 2023-09-07

**Authors:** Federica De Muzio, Fabio Pellegrino, Roberta Fusco, Salvatore Tafuto, Mariano Scaglione, Alessandro Ottaiano, Antonella Petrillo, Francesco Izzo, Vincenza Granata

**Affiliations:** 1 Department of Medicine and Health Sciences “V. Tiberio”, University of Molise, 86100 Campobasso, Italy; demuziofederica@gmail.com; 2 Radiology Division, S. Bonifacio Hospital, 37047 Verona, Italy; 3 Medical Oncology Division, Igea SpA, 80013 Napoli, Italy; r.fusco@igeamedical.com; 4 Unit of Sarcomi e Tumori Rari, Istituto Nazionale Tumori, IRCCS, Fondazione G. Pascale, 80131 Naples, Italy; s.tafuto@istitutotumori.na.it; 5 Department of Medical, Surgical and Experimental Sciences, University of Sassari, 07100 Sassari, Italy; 6 Unit for Innovative Therapies of Abdominal Metastastes, Istituto Nazionale Tumori, IRCCS, Fondazione G. Pascale, 80131 Naples, Italy; a.ottaiano@istitutotumori.na.it; 7 Division of Radiology, Istituto Nazionale Tumori, IRCCS, Fondazione G. Pascale, 80131 Naples, Italy; a.petrillo@istitutotumori.na.it; 8 Division of Hepatobiliary Surgery, Istituto Nazionale Tumori, IRCCS, Fondazione G. Pascale, 80131 Naples, Italy

**Keywords:** neuroendocrine neoplasms, imaging, computed tomography, magnetic resonance imaging, radiomics

## Abstract

Neuroendocrine neoplasms (NENs) are a group of lesions originating from cells of the diffuse neuroendocrine system. NENs may involve different sites, including the gastrointestinal tract (GEP-NENs). The incidence and prevalence of GEP-NENs has been constantly rising thanks to the increased diagnostic power of imaging and immuno–histochemistry. Despite the plethora of biochemical markers and imaging techniques, the prognosis and therapeutic choice in GEP-NENs still represents a challenge, mainly due to the great heterogeneity in terms of tumor lesions and clinical behavior. The concept that biomedical images contain information about tissue heterogeneity and pathological processes invisible to the human eye is now well established. From this substrate comes the idea of radiomics. Computational analysis has achieved promising results in several oncological settings, and the use of radiomics in different types of GEP-NENs is growing in the field of research, yet with conflicting results. The aim of this narrative review is to provide a comprehensive update on the role of radiomics on GEP-NEN management, focusing on the main clinical aspects analyzed by most existing reports: predicting tumor grade, distinguishing NET from other tumors, and prognosis assessment.

## 1. Introduction

Neuroendocrine neoplasms (NENs) are tumors with significant heterogeneity and complex clinical behavior that originate from cells of the diffuse neuroendocrine system [1].

**Figure 1:**
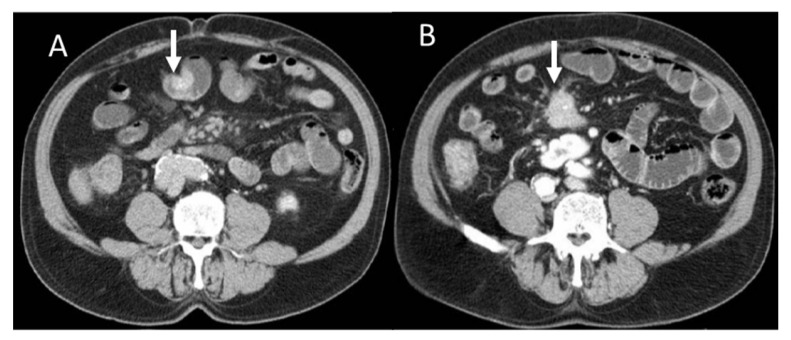
Portal CT evaluation of small bowel NEN (arrow in (**A**)): intraluminal enhanced polypoid lesion (arrow) with desmoplastic reaction (arrow in (**B**)).

**Figure 2:**
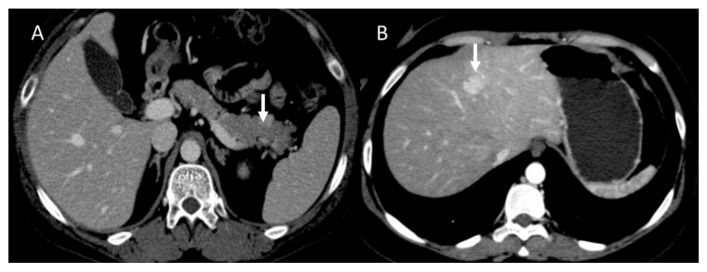
CT evaluation during portal phase of pancreatic NEN ((**A**): arrow) with liver metastasis ((**B**): arrow).

NENs could be divided into two categories: neuroendocrine tumors (NETs) and neuroendocrine carcinomas (NECs). NETs are well-differentiated, slow growing, but potentially malignant lesions, while NECs are poorly differentiated and highly aggressive carcinomas [2]. NENs may involve many different sites, and in two thirds of cases, may arise in the gastro–entero–pancreatic tract (GEP). Of these, nearly 50% are intestinal (Figure 1) and 30% pancreatic [1] (Figure 2). Among all malignancies in this district, NENs are considered rare (1.0–1.5% of all GEP tumors) [3,4], but their incidence and prevalence has been constantly rising (currently estimated at 3.0–5.2 cases per 100,000 people per year, and 35 cases per 100,000 people per year, respectively) [5] thanks to the increased power of detection through imaging and immuno–histochemistry.

The majority of GEP-NENs (>95%) occurs in sporadic forms, while 5% of cases are part of a polydistrict syndrome, such as multiple endocrine neoplasm type 1 (MEN1), neurofibromatosis type 1 (NF1), and von Hippel–Lindau syndrome (VHL) [6,7]. GEP-NENs could release metabolically active hormones and amines, leading to hypersecretion-specific clinical signs and symptoms, which could facilitate the diagnosis. In this scenario, the diagnosis of functional lesions is clinical, and diagnostic tools are employed to localize the lesion and to stage. Unfortunately, intestinal NENs secrete hormones in only 20%, and pancreatic NENs are functional in only 10–30% [6,7]. So, in these indolent lesions, the presence of non-specific symptoms can cause a delayed diagnosis, with lesions identified in an advanced stage, often for complications due to the primary tumor (bowel obstruction, mass effect) or metastases (especially in the liver) [6,7]. Consequently, an accurate diagnosis requires a multidisciplinary assessment, including a morphological and functional imaging evaluation.

**Figure 3:**
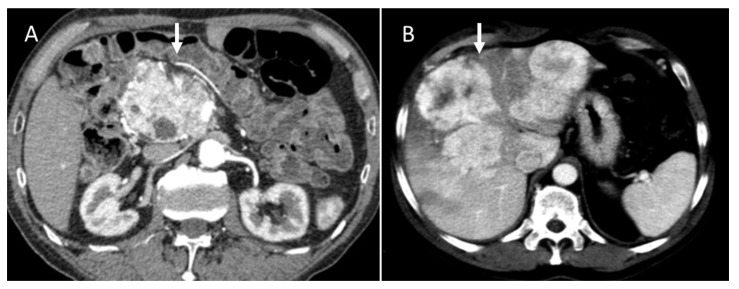
CT evaluation during arterial phase of pancreatic NEN ((**A**): arrow) with liver metastases ((**B**): arrow).

The main prognostic factors for GEP-NETs are the primary site, with a poorer prognosis in the pancreatic location (PNET) (Figure 3); the TNM stage; and the histopathological classification according to the World Health Organization (WHO), which includes the morphological and proliferative criteria based on the Ki67 index [1]. An additional positive prognostic factor is the expression of the somatostatin receptor, which is the basis of imaging diagnosis on Gallium-68 DOTA-TATE Positron Emission Tomography/Computed Tomography (PET/CT) and somatostatin analog (SSA) therapy, including octreotide acetate and peptide receptor radionuclide therapy (PRRT) (such as lutetium Lu 177 dotatate) [6,7].

The prognosis and therapeutic choice in NETs still represents a challenge, mainly due to the great heterogeneity of this family of tumors with varied clinical behavior [8,9].

Regarding imaging evaluation, even if the limits of conventional morphological studies are well recognized, technique optimization, such as technological improvements, with the use of new sequences in magnetic resonance imaging (MRI) have allowed for obtaining a high sensitivity in detection rate. Additionally, conventional imaging allows local staging in order to identify patients fit for surgery, considering that surgical resection, according to ESMO guidelines, is the front line of treatment for many lesions.

According to the guidelines of the European Neuroendocrine Tumor Society (ENETS), contrast-enhanced neck–thorax–abdomen and pelvis CT, which should include a three-phase liver assessment, is suggested during detection, stage, treatment response evaluation, and surveillance phases. Contrast-enhanced MRI, including diffusion-weighted imaging (DWI), is suggested for hepatic, pancreatic, head, and bone evaluation. Ultrasonography (US) with contrast-enhanced ultrasound (CEUS) is suggested as a solution in liver lesions and is a useful tool during surgical resection. In addition, for jejunum and ileum tumors, CT enteroclysis is the diagnostic tool that should be chosen, while in liver and pancreatic lesion assessment, MRI should be chosen. CT permits a vascular assessment in the pre-surgical phase.

With regard to functional evaluation, 68Ga/64Cu-DOTA-somatostatin analogue PET-CT showed the higher sensitivity in NEN detection, and according to ENETS, this tool should be chosen to localize the disease in non-insulinoma pancreatic lesions. In addition, the European Society of Medical Oncology (ESMO) guidelines suggest the use of PET with [18F] fluoro–deoxy–glucose (FDG) as an optional tool in NEN assessment. However, this tool is useful for G3 and high G2 lesions, considering the higher glucose metabolism and the lesser Somatostatin receptor (SSTR) expression compared to low-grade lesions.

The concept that biomedical images contain information about tissue heterogeneity and complex pathological processes is now well established [10,11]. From this substrate comes the idea of radiomics, a quantitative approach to medical imaging that aims to decode the interrelationships between pixels that are invisible to the human eye. From each voxel of a selected tissue region on different imaging techniques, hundreds of features could be extracted and analyzed with multiple post-processing methods and software [12,13]. The ultimate goal is to create faster and more reliable support systems for assisting clinical decision-making and to improve the quality of care [12,13,14,15,16,17,18,19,20,21,22,23,24,25,26,27,28,29,30,31,32,33,34,35,36,37]. The radiomics process includes image acquisition and segmentation, feature extraction and qualification, feature selection, analysis, and classification. The image data are provided by radiological modalities, while feature segmentation consists of the isolation of volume interest regarding the target zone to analyze.

The features were extracted (feature extraction) by means of several tools, such as the Pyradiomics tool 3D Slicer platform (https://pyradiomics.readthedocs.io/en/latest (accessed on 1 January 2021)).

Radiomic features can be divided into six groups: size and shape-based features, image intensity histogram descriptors, image–voxel relationship descriptors, size zone matrix (SZM)-derived plots, and neighborhood grayscale difference matrix (NGTDM), and filtered image textures and fractal features. Two-dimensional and three-dimensional feature extraction can be used to calculate individual feature values for a region of interest (“segment-based”) or to generate feature maps (“voxel-based”). Feature extraction should be carried out according to the Image Biomarker Standardization Initiative (IBSI), an independent international collaboration working to standardize feature extraction.

Due to its huge variety, feature reductions need to be implemented to eliminate redundant information, unstable and unreproducible features that could lead to spurious results and unrepeatable patterns. After selecting the important features, it is essential to analyze the chosen data by first comparing the features with each other to find out if they have any information in common and to reveal what it means when they all occur at the same time. Other methods of testing are supervised or unsupervised testing. The algorithm needs to recognize the correlations between images and features.

Several steps are required to build an integrated radiomics database. Imaging data should be exported from clinics, which is already complex because patient information is very sensitive and governed by privacy laws. Furthermore, the exported data must not lose their integrity when compressed so that the database only incorporates data of the same quality. Furthermore, the integration of clinical and molecular data is important for various clinical outcomes. A large image storage location is required in this case.

Computational analysis has achieved promising results in several oncological settings [38,39,40,41,42,43,44,45,46,47,48,49,50,51,52,53,54,55,56,57,58], and the use of radiomics in different types of GEP-NET is growing in this field of research [18,59,60], yet with conflicting results.

The aim of this narrative review is to provide a comprehensive update on the role of radiomics on GEP-NETs management, focusing on the main clinical aspects analyzed by most existing reports: predicting tumor grade in PNETs, distinguishing PNET from other tumors, and prognosis assessment.

## 2. Tumor Classification

The differential diagnosis among types of pancreatic cancers as well as benign diseases could be difficult [61,62,63].

A proper lesion characterization allows us to choose the best treatment according to patient status. In fact, although surgical resection is the only curative treatment in pancreatic ductal adenocarcinomas (PDAC), when the lesion is detected in an early phase, with regard to PNEN, surgical treatment should be in G1 and G2 NEN, although several authors suggested a watch-and-wait approach for asymptomatic non-functional lesions <2 cm. However, surgery is the front line treatment in young patients in cases of local invasiveness and in the presence of functioning lesions. In this scenario, the necessity of a proper lesion characterization is evident, although biopsy remains an invasive approach and problematic for small and deep lesions.

**Figure 4:**
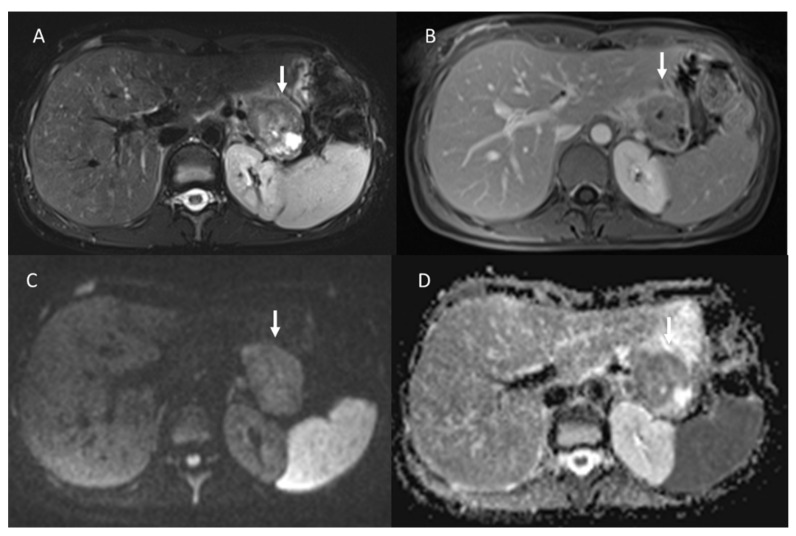
MRI assessment of pancreatic NEN. In T2-W (**A**) sequence, the lesion (arrow) is inhomogeneous and hypo–hyperintense due to fibrotic component. This feature is evident in late phase (**B**) of contrast study (arrow). In DWI ((**C**): b800 s/mm^2^), the lesion (arrow) shows restricted diffusion with hypointense signal in ADC map (arrow in (**D**)).

In the presence of unclear imaging results in the diagnosis of PNEN, CT radiomics can be helpful in determining the type of tumor. In fact, PNECs in the portal venous phase on CT with contrast media tend to show greater uniformity and less entropy than PDACs in the absence of significant differences in these parameters in the arterial phase, kurtosis, and asymmetry (heterogeneity parameters) [63]. The cut-off value of 0.34 has been suggested for uniformity in differentiating NEC from PDAC with a sensitivity and specificity of 79% and 65%, and a cut-off value of 1.89 for entropy with a sensitivity of 74% and a specificity of 70% [63]. Radiomics is also particularly useful in differentiating atypical PNENs that show hypovascularization in the CT arterial phase from PDACs [64]. In fact, the latter have a greater skewness (which is a measure of heterogeneity) than atypical PNETs with lower mean, median, fifth, tenth, and twenty-fifth percentiles on the entire tumor structure by contrast-enhanced CT (CECT) compared to those of atypical PNET. The authors suggest that this result could be linked to a greater cystic necrosis and degeneration. The fifth percentile alone or combined with asymmetry was capable in differentiating the two groups with high sensitivity: when considered alone, a sensitivity of 96% and a moderate specificity of 64% were reached, while in combination, a sensitivity of 90% and a specificity of 80% were reached [64]. In another study, it emerged that a model of seven radiomic characteristics can differentiate between atypical PDAC and PNET with greater sensitivity and specificity than the model based on clinical radiological parameters alone [65]. Neuroendocrine tumors can be differentiated from adenocarcinomas by histogram analysis performed in DWI of apparent diffusion coefficient (ADC) values on MRI (Figure 4). PDACs showed greater kurtosis (heterogeneity marker) and skewness in ADC histogram analysis on ADC_400_ (b value 0–400 s/mm^2^) and ADC_800_ (b value 0–800 s/mm^2^) than PNET tumors, while neuroendocrine tumors, as seen in the analysis of the CT texture, have a significantly lower entropy, regardless of the b value [66].

In addition to PDAC, radiomics seems capable of identifying other pathological entities that go into differential diagnosis with the PNETs, requiring different diagnostic-therapeutic pathways.

Among all machine-learning classifiers on CT-based radiomics analysis, Random Forest (RF) seems to be an excellent classification algorithm for differentiating PNETs from pancreatic cystadenomas with high accuracy, sensitivity, and specificity values (0.983, 0.980, and 0.986, respectively) [67]. Radiomic features of MRI may discriminate PNENs from pancreatic pseudopapillary neoplasms (SPTs), which often have similar imaging features, especially when small [68]. Furthermore, Shi et al. developed a radiomics model that incorporated the sex and age of patients and the radiomics signature of the tumor with excellent discrimination performance for diagnosing SPTs and PNETs with an area under curve (AUC) of 0.97 and 0.86 in a primary and validation cohort, compared to median diffusivity (MD) and median kurtosis (MK) diagnostic performance (area under curve of 0.71 and 0.65, respectively) [69].

More generally, radiomic models outperformed the clinical radiological model in discrimination against both PDACs and other lesions, reaching even higher values when a holistic model incorporated clinical and radiomics features was developed [68,69,70,71,72,73,74,75,76,77,78,79,80,81,82,83,84,85,86,87,88].

### Tumor Grading

Most GEP-NEN radiomics studies focus on the evaluation of tumor grading in pNENs [89].

PNENs, according to the 2010 WHO classification system, were divided into three grades for their mitotic count and Ki-67 index: G1, low-grade (mitotic count < 2 per 10 high power fields (HPFs) and/or a Ki67 index of <3%); G2, intermediate-grade (mitotic count 2–20 per 10 HPF and/or a Ki67 index of 3–20%); and G3, high-grade (mitotic count > 20 per 10 HPF and/or a Ki67 index of >20%). These are also called poorly differentiated neuroendocrine carcinomas (pNECs). The 2017 WHO update reports that a subset of pNET G3 was identified with survival times shorter than those for pNET G1 or G2 but longer than typically described for pNEC (5-year survival rates varied from 60 to 100% for G1/2 and 16 to 29% for G3 pNEN) [90]. Treatment decisions for patients with pNETs are usually guided after staging the disease assessment. According to the European Society for Medical Oncology (ESMO) guidelines [91], treatment planning for pNEN G1 and G2 may involve only surgical resection with a sparing approach, while for more undifferentiated lesions, systemic therapy may be required [91]. So, the ability to distinguish the tumor grade of NETs preoperatively could have a huge impact on patient management and several radiomics features demonstrated to correlate with this prognostic factor [92].

Entropy, uniformity, and kurtosis extracted by CT imaging were significantly different between G1/2 tumors and G3 tumors [93,94,95,96]. Moreover, at high sigmoid levels, kurtosis was significantly different between G1 and G2 tumors. In addition, entropy was the most sensitive parameter to differentiate between G1/2 PNET and G3 PNEC, obtaining a sensitivity of 91% and a specificity of 85%. In another study, the entropy resulted in the only feature capable of predicting tumor grade (accuracy = 65%) [97]. Among the different variables, similar to CT radiomics, entropy resulted in the best predictor of tumor grade and aggressiveness, even on MRI images [93,98,99,100,101,102,103,104,105]. In addition, an increase of asymmetry and kurtosis values was shown with an increase of tumor grade [93]. The entropy of ADC was significantly higher in G2 and G3 tumors than in well-differentiated G1 (cut-off of 6.6) tumors, with a sensitivity and specificity of 83.3% and 61.1%, respectively, and an AUC of 0.75 [98].

Studies with a significant Radiomics Quality Score (RQS) and application of several machine-learning algorithms demonstrated a higher-order feature correlation with a prediction of pNET tumor grade [106,107]. On CT imaging, a higher value of “GLSZM Small Area High Gray-Level Emphasis” on the portal venous phase was recorded in G1 (0.80  ±  0.90) compared to G2/3 tumors (− 0.47  ±  0.86; *p*  < 0 .05) [107], while several features, such as GLCM_Homogeneity and GLCM_Entropy_log10, were selected from RF and discriminative of G3 tumors [106].

Some authors developed prediction models for PNET grading [108,109,110,111] built from combinations of multiple features extracted from radiological images, with encouraging results. In one study [108], researchers formulated a radiomic score based on four high-order selected features (compactness1.shape.original, LongRunHighGrayLevelEmphasis.GLRLM.original, SmallAreaHighGrayLevelEmphasis.GLSZM.squareroot, and skewness.firstorder.wavelet.LLH) extracted from the portal venous phase, with an overall high accuracy in tumor grading even in small pNET lesions (AUC = 0.86 for all PNETs; AUC = 0.81 for PNETs ≤ 2 cm). In contrast, Choi et al. [109] constructed a logistic regression model with the radiologic features of 66 pNET patients (45 patients with G1 tumors and 21 patients with G2/3 tumors) and the textural features of the arterial and venous phases leading to CT being capable of identifying the most undifferentiated lesions; this model is better suited for tumor grade determination (AUC = 0.77) than simple CT (AUC = 0.68). In line with these results, Canellas et al. [97] established a logistic regression model that was able to predict grading with an accuracy of 79.3%, considering entropy in the portal phase with CT imaging features of pNET (tumor diameter, vascular invasion, pancreatic duct dilation, lymphatic metastasis). However, higher performance is recorded from holistic models based on radiomics, clinical, and radiological features. Gu et al. [110], in a multicenter study, proposed a clinical/radiomic model, including tumor margin and the fusion radiomic signature from the arterial and portal venous phase CT images, demonstrating an optimal performance in both training and validation cohorts (accuracy value of 89.4% and 76.5%, respectively). Similarly, Liang et al. [111] combined clinical radiological data and CT radiomic features in a model, which resulted in being able to differentiate tumor grading in both training and validation sets with high accuracy (training set: AUC = 0.907; validation set: AUC = 0.891).

## 3. Results

An appealing target of radiomics in an oncological setting is the possibility to successfully predict a prognosis through the computational analysis of multiple preoperative parameters [39,40,41,42]. The ultimate purpose is to obtain individualized clinical decision-making and to increase patients’ survival rates [112,113,114,115].

Radiomics consists of the extraction of multiple features from medical images using mathematical algorithms. These features may have the potential to uncover tumor patterns and characteristics that cannot be appreciated with the naked eye. Volumes of raw data produced by CT, MRI, PET/CT, or even PET/MRI are used to find different pixel/voxel characteristics that can be useful for predicting prognoses and therapeutic responses for various types of cancer, thus providing valuable insights for therapy and prognosis [39,40,41,42].

The presence of lymph node metastasis plays a substantial prognostic role in pancreatic and small bowel (SB) NETs. Changes in the cytokine milieu, the expansion of immunosuppressive cell lineages, and an increase in lymphangiogenesis all contribute to the seeding, survival, and subsequent expansion of tumor cells within the lymph node. The surgical resection of tumor-draining lymph nodes has come under scrutiny, especially in more superficial tumors, where an extended lymph node dissection is often associated with substantial morbidity for patients [6,7]. In SB-NETs, however, regional mesenteric lymphadenectomy has been associated with improved survival. Nonetheless, the extent of lymphadenectomy has come under question. Patients with too few resected lymph nodes can be understaged, whereas the resection of a greater number of lymph nodes may enable the detection of clinically silent lymph node metastases. It is known that the limits of imaging in evaluating the real involvement of nodes is a diameter <15 mm; however, radiomics is in the early stages with regard to this feature [18].

So, several authors have focused on the performance of radiomics in predicting the recurrence and long-term clinical outcome in GEP-NETs.

### 3.1. Prediction of Survival

Chen et al. [116] developed and validated a computed tomography (CT)-based method to predict the efficacy of sunitinib in patients with pancreatic NET. Tumor shrinkage > 10% at the first follow-up after sunitinib treatment was significantly associated with longer progression-free survival (PFS; *p* < 0.001) and was used as the primary treatment outcome. The authors then developed a radiomic signature that showed significantly higher AUC in the training (0.915) and validation (0.770) sets than the ratio of CT values. The proposed radiomics model accurately predicted tumor shrinkage and progression-free survival in pancreatic NET patients treated with sunitinib, and might help select patients suitable for sunitinib treatment.

Regarding the 5-year survival status, a machine-learning algorithm and DL demonstrated excellent results (AUC ranging from 0.87 to 0.90), as highlighted in two studies [116,117] on a large population sample with external validation cohorts. These reports [116,117] are developed on data retrospectively extracted from the large database of the Surveillance, Epidemiology, and End Results (SEER) registry, using the AJCC seventh staging system as the gold standard for prognosis assessment, giving these conclusions a certain reliability. Testing survival rates among GEP NET, Werner et al. [118] observed that entropy was independently associated with PFS and overall survival (OS), while skewness resulted in being independently associated with OS.

### 3.2. Prediction of Tumor Aggressiveness

Martini et al. [119] further outlined an inverse correlation between entropy and OS and a direct correlation between skewness and OS in a radiomics model based on CT imaging.

**Figure 5:**
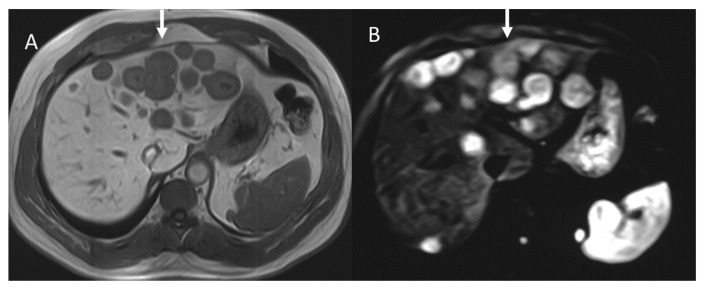
MRI evaluation of liver pNEN metastases. Morphological evaluation ((**A**): T1-W image) show hypointense lesions (arrow); in DWI ((**B**): b800 s/mm^2^), lesions show restricted signal.

In contrast, in 68GaSSA PET/CT radiomics analysis, a higher entropy value resulted in a better association with PFS and OS, [120] in patients treated with PRRT. The entropy and kurtosis of the ADC were found to be higher in aggressive tumors, especially those with vascular invasion, lymph node disease, and liver metastases [98] (Figure 5). Although several parameters were significant for several aggressivity markers, kurtosis was found to be the best parameter in the identification of vascular invasion, showing an AUC of 0.763 using a cut-off value equal to 4.13, and in the identification of distant metastases, showing an AUC of 0.820 using a cut-off value of 3.64 [98].

### 3.3. Prediction of Recurrences

In terms of disease recurrence appraisal, clinical data may significantly impact when added to radiomic features, as shown by one study [121] on 225 GP-NETs. Considering radiomics scores and clinical pathological factors (age, Ki-67 index, tumor pathological type, tumor primary site, and TNM stage), An et al. [121] were able to properly assess the non-recurrent group and the recurrent group more accurately than the individual models, which analyzed independently (combined model with an AUC of 0.824; clinical data model with an AUC of 0.786, and radiomics model with an AUC of 0.712).

Different methods for recurrence assessment in GP-NENs [92] focused on KI67, which is an important prognostic indicator. In recent years, several deep-learner-based approaches have emerged to evaluate Ki-67 indices [122]. Vesterinen et al. [123] built a deep-learning-based Ki-67 proliferation index PI algorithm (KAI) that objectively calculates Ki-67 PI, while Govind et al. [124] were able to generate a comprehensive view of the tumor via a Ki-67 index-based heatmap. Radiomics models [123,124] showed an excellent correspondence with pathologists’ assessment on postoperative specimens or preoperative biopsy, which are hampered by being invasive and not automatically representative of the whole lesion. The possibility to capture the complex intratumoral heterogeneity is an important tool in intercepting the tumor’s overall biological behavior [125,126,127,128,129]. From a clinical perspective, these open up the possibility to select patients and guide the right treatment choice, allowing a more personalized approach.

## 4. Prospects and Limits

Radiomics has already been demonstrated to improve tumor diagnosis, grading and staging, evaluation of responses to therapy, and prognosis prediction [130,131,132,133,134,135]. Machine-learning and deep-learning algorithms have supported fast processes of quantitative data analysis, further increasing the application of radiomics in oncological settings [136,137,138].

Additionally, radiogenomics can be used to create imaging biomarkers that can identify the genomics of a disease without the use of a biopsy, and various techniques are used to find statistically significant correlations between MRI, CT, and PET-imaging features and disease genomics [136,137,138].

The possibility to obtain biological information without an invasive approach is particularly attractive [139,140,141,142,143,144,145]. GEP-NETs are rare tumors with varied biological behavior [1,2,3]. Intercepting radiomics features useful in the management of these clusters of patients is of great scientific interest.

As noted in this review, most studies concerned the prediction of tumor grade [93,94,95,96,97,98,99,100,101,102,103,104,105,106,107,108,109,110,111]. The use of radiomics in the prediction of histological and genetic characteristics in different types of PNEN-evaluating images, both quantitatively and objectively, with texture analysis is growing in this field of research. From each voxel of a selected tissue region, hundreds of first-order quantitative data, such as entropy, and/or second-order characteristics, such as gray level, are extracted and analyzed with different post-processing methods and software. First-order features linked to increased heterogeneity, such as entropy and kurtosis [93,98,99,100,101,102,103,104], resulted in more undifferentiated tumors [93,94,95,96,97,98]. These data could be tested on GEP-NETs other than pancreatic type. Entropy was also frequently reported as a prognosis and recurrence predictor [45,47], and might be worth further exploring. First-order statistics were also the best predictors for distinguishing PNETs from PDACs [63,64]. Ultimately, the best performance was achieved when radiomics features were combined with clinical features [69,110,111,121]. All this evidence still needs to be reproduced in a larger cohort of patients and in a prospective manner to ensure reliability.

The standardization and retrospective nature of these studies are critical issues [145,146,147,148,149,150,151,152,153,154,155,156,157]. Of note, most of the available reports are focused on pancreatic NETs, and almost completely lacking any assessment of prognosis and long-term outcomes [18]. This is probably related to the rarity of these lesions and the difficulty of recruiting patients, which is reflected in research with small samples and external validation sets that are not always available. A separate validation dataset is an important element to avoid overfitting, which is another critical issue in radiomics surveys [146,147,148,149,150,151,152,153,154,155,156,157,158]. Additionally, current radiomics methods are limited to the use of single image sets for radiomics feature extraction and may not be able to capture the true features of the underlying tissues in the high-dimensional multiparameter-imaging space.

Consequently, in most cases, reports do not meet quality standards, and the predictive power of radiomics may be overestimated.

## 5. Conclusions

Although the evidence is promising, radiomics in GEP-NETs is still in its early stages. It has been preliminarily demonstrated that several radiomic features are correlated with some prognostic factors in GEP-NETs, but the combined clinical and radiomic models appear to achieve greater accuracy in lesion assessment. Radiomic models outperformed clinic-radiological model, both in discrimination against PDACs and other lesions, reaching even higher values when a holistic model incorporated clinical and radiomics features is developed. Regarding the 5-year survival status, Machine learning algorithm and DL demonstrated excellent results so as in tumor aggressiveness and recurrence. However, in most cases, the reports do not meet quality standards, and the predictive power of radiomics may be overestimated.

In the future, noninvasive predictive models may assist clinicians to optimize tailored therapeutic strategies for each patient with GEP-NETs in daily practice.

## Data Availability

All data are reported in the manuscript.

## References

[1] Fernandez C.J., Agarwal M., Pottakkat B., Haroon N.N., George A.S., Pappachan J.M. (2021). Gastroenteropancreatic neuroendocrine neoplasms: A clinical snapshot. World J. Gastrointest. Surg..

[2] Yao J.C., Hassan M., Phan A., Dagohoy C., Leary C., Mares J.E., Abdalla E.K., Fleming J.B., Vauthey J.N., Rashid A. (2008). One hundred years after “carcinoid”: Epidemiology of and prognostic factors for neuroendocrine tumors in 35,825 cases in the United States. J. Clin. Oncol..

[3] Ito T., Masui T., Komoto I., Doi R., Osamura R.Y., Sakurai A., Ikeda M., Takano K., Igarashi H., Shimatsu A. (2021). JNETS clinical practice guidelines for gastroenteropancreatic neuroendocrine neoplasms: Diagnosis, treatment, and follow-up: A synopsis. J. Gastroenterol..

[4] Cheung V.T.F., Khan M.S. (2015). A guide to midgut neuroendocrine tumours (NETs) and carcinoid syndrome. Frontline Gastroenterol..

[5] Dasari A., Shen C., Halperin D., Zhao B., Zhou S., Xu Y., Shih T., Yao J.C. (2017). Trends in the Incidence, Prevalence, and Survival Outcomes in Patients with Neuroendocrine Tumors in the United States. JAMA Oncol..

[6] Granata V., Fusco R., Setola S.V., Castelguidone E.L.D., Camera L., Tafuto S., Avallone A., Belli A., Incollingo P., Palaia R. (2019). The multidisciplinary team for gastroenteropancreatic neuroendocrine tumours: The radiologist’s challenge. Radiol. Oncol..

[7] Chiti G., Grazzini G., Cozzi D., Danti G., Matteuzzi B., Granata V., Pradella S., Recchia L., Brunese L., Miele V. (2021). Imaging of Pancreatic Neuroendocrine Neoplasms. Int. J. Environ. Res. Public Health.

[8] Walter D., Harter P.N., Battke F., Winkelmann R., Schneider M., Holzer K., Koch C., Bojunga J., Zeuzem S., Hansmann M.L. (2018). Genetic heterogeneity of primary lesion and metastasis in small intestine neuroendocrine tumors. Sci. Rep..

[9] Yang Z., Tang L.H., Klimstra D.S. (2011). Effect of tumor heterogeneity on the assessment of Ki67 labeling index in well-differentiated neuroendocrine tumors metastatic to the liver: Implications for prognostic stratification. Am. J. Surg. Pathol..

[10] Granata V., Fusco R., De Muzio F., Cutolo C., Setola S.V., Grassi R., Grassi F., Ottaiano A., Nasti G., Tatangelo F. (2022). Radiomics textural features by MR imaging to assess clinical outcomes following liver resection in colorectal liver metastases. Radiol. Med..

[11] Granata V., Fusco R., Setola S.V., De Muzio F., Dell’Aversana F., Cutolo C., Faggioni L., Miele V., Izzo F., Petrillo A. (2022). CT-Based Radiomics Analysis to Predict Histopathological Outcomes Following Liver Resection in Colorectal Liver Metastases. Cancers.

[12] Golse N., Nunez J., Mazzotta A., Cano L., Bergeat D., Sulpice L., Jeddou H., Abdelrafee A., Sa Cunha A., Cherqui D. (2020). Personalized Preoperative Nomograms Predicting Postoperative Risks after Resection of Perihilar Cholangiocarcinoma. World J. Surg..

[13] Davnall F., Yip C.S., Ljungqvist G., Selmi M., Ng F., Sanghera B., Ganeshan B., Miles K.A., Cook G.J., Goh V. (2012). Assessment of tumor heterogeneity: An emerging imaging tool for clinical practice?. Insights Imaging.

[14] Argalia G., Tarantino G., Ventura C., Campioni D., Tagliati C., Guardati P., Kostandini A., Marzioni M., Giuseppetti G.M., Giovagnoni A. (2021). Shear wave elastography and transient elastography in HCV patients after direct-acting antivirals. Radiol. Med..

[15] Giovagnoni A. (2021). A farewell from the “old” Editor-in-Chief. Radiol. Med..

[16] Cicero G., Mazziotti S., Silipigni S., Blandino A., Cantisani V., Pergolizzi S., D’Angelo T., Stagno A., Maimone S., Squadrito G. (2021). Dual-energy CT quantification of fractional extracellular space in cirrhotic patients: Comparison between early and delayed equilibrium phases and correlation with oesophageal varices. Radiol. Med..

[17] Stefanini M., Simonetti G. (2022). Interventional Magnetic Resonance Imaging Suite (IMRIS): How to build and how to use. Radiol. Med..

[18] Staal F.C.R., Aalbersberg E.A., van der Velden D., Wilthagen E.A., Tesselaar M.E.T., Beets-Tan R.G.H., Maas M. (2022). GEP-NET radiomics: A systematic review and radiomics quality score assessment. Eur. Radiol..

[19] Nakamura Y., Higaki T., Honda Y., Tatsugami F., Tani C., Fukumoto W., Narita K., Kondo S., Akagi M., Awai K. (2021). Advanced CT techniques for assessing hepatocellular carcinoma. Radiol. Med..

[20] Fusco R., Setola S.V., Raiano N., Granata V., Cerciello V., Pecori B., Petrillo A. (2022). Analysis of a monocentric computed tomography dosimetric database using a radiation dose index monitoring software: Dose levels and alerts before and after the implementation of the adaptive statistical iterative reconstruction on CT images. Radiol. Med..

[21] Silva M., Picozzi G., Sverzellati N., Anglesio S., Bartolucci M., Cavigli E., Deliperi A., Falchini M., Falaschi F., Ghio D. (2022). Low-dose CT for lung cancer screening: Position paper from the Italian college of thoracic radiology. Radiol. Med..

[22] Ierardi A.M., Stellato E., Pellegrino G., Bonelli C., Cellina M., Renzulli M., Biondetti P., Carrafiello G. (2022). Fluid-dynamic control microcatheter used with glue: Preliminary experience on its feasibility and safety. Radiol. Med..

[23] Park S.H., Kim Y.S., Choi J. (2021). Dosimetric analysis of the effects of a temporary tissue expander on the radiotherapy technique. Radiol. Med..

[24] Bozkurt M., Eldem G., Bozbulut U.B., Bozkurt M.F., Kılıçkap S., Peynircioğlu B., Çil B., Lay Ergün E., Volkan-Salanci B. (2021). Factors affecting the response to Y-90 microsphere therapy in the cholangiocarcinoma patients. Radiol. Med..

[25] Giurazza F., Cionfoli N., Paladini A., Vallone M., Corvino F., Teodoli L., Moramarco L., Quaretti P., Catalano C., Niola R. (2022). PHIL^®^ (precipitating hydrophobic injectable liquid): Retrospective multicenter experience on 178 patients in peripheral embolizations. Radiol. Med..

[26] Falcinelli L., Mendichi M., Chierchini S., Tenti M.V., Bellavita R., Saldi S., Ingrosso G., Reggioli V., Bini V., Aristei C. (2021). Pulmonary function in stereotactic body radiotherapy with helical tomotherapy for primary and metastatic lung lesions. Radiol. Med..

[27] Arslan A., Aktas E., Sengul B., Tekin B. (2021). Dosimetric evaluation of left ventricle and left anterior descending artery in left breast radiotherapy. Radiol. Med..

[28] Barra S., Guarnieri A., di Monale EBastia M.B., Marcenaro M., Tornari E., Belgioia L., Magrini S.M., Ricardi U., Corvò R. (2021). Short fractionation radiotherapy for early prostate cancer in the time of COVID-19: Long-term excellent outcomes from a multicenter Italian trial suggest a larger adoption in clinical practice. Radiol. Med..

[29] Cellini F., Di Franco R., Manfrida S., Borzillo V., Maranzano E., Pergolizzi S., Morganti A.G., Fusco V., Deodato F., Santarelli M. (2021). Palliative radiotherapy indications during the COVID-19 pandemic and in future complex logistic settings: The NORMALITY model. Radiol. Med..

[30] Lancellotta V., Del Regno L., Di Stefani A., Fionda B., Marazzi F., Rossi E., Balducci M., Pampena R., Morganti A.G., Mangoni M. (2022). The role of stereotactic radiotherapy in addition to immunotherapy in the management of melanoma brain metastases: Results of a systematic review. Radiol. Med..

[31] Hussein M.A.M., Cafarelli F.P., Paparella M.T., Rennie W.J., Guglielmi G. (2021). Phosphaturic mesenchymal tumors: Radiological aspects and suggested imaging pathway. Radiol. Med..

[32] Fushimi Y., Yoshida K., Okawa M., Maki T., Nakajima S., Sakata A., Okuchi S., Hinoda T., Kanagaki M., Nakamoto Y. (2022). Vessel wall MR imaging in neuroradiology. Radiol. Med..

[33] Granata V., Simonetti I., Fusco R., Setola S.V., Izzo F., Scarpato L., Vanella V., Festino L., Simeone E., Ascierto P.A. (2022). Management of cutaneous melanoma: Radiologists challenging and risk assessment. Radiol. Med..

[34] Granata V., Fusco R., Setola S.V., Galdiero R., Maggialetti N., Silvestro L., De Bellis M., Di Girolamo E., Grazzini G., Chiti G. (2023). Risk Assessment and Pancreatic Cancer: Diagnostic Management and Artificial Intelligence. Cancers.

[35] Granata V., Fusco R., Barretta M.L., Picone C., Avallone A., Belli A., Patrone R., Ferrante M., Cozzi D., Grassi R. (2021). Radiomics in hepatic metastasis by colorectal cancer. Infect. Agents Cancer.

[36] Granata V., Grassi R., Fusco R., Setola S.V., Belli A., Ottaiano A., Nasti G., La Porta M., Danti G., Cappabianca S. (2021). Intrahepatic cholangiocarcinoma and its differential diagnosis at MRI: How radiologist should assess MR features. Radiol. Med..

[37] Fusco R., Granata V., Sansone M., Rega D., Delrio P., Tatangelo F., Romano C., Avallone A., Pupo D., Giordano M. (2021). Validation of the standardized index of shape tool to analyze DCE-MRI data in the assessment of neo-adjuvant therapy in locally advanced rectal cancer. Radiol. Med..

[38] Aerts H.J., Velazquez E.R., Leijenaar R.T., Parmar C., Grossmann P., Carvalho S., Bussink J., Monshouwer R., Haibe-Kains B., Rietveld D. (2014). Decoding tumour phenotype by noninvasive imaging using a quantitative radiomics approach. Nat. Commun..

[39] Santone A., Brunese M.C., Donnarumma F., Guerriero P., Mercaldo F., Reginelli A., Miele V., Giovagnoni A., Brunese L. (2021). Radiomic features for prostate cancer grade detection through formal verification. Radiol. Med..

[40] Benedetti G., Mori M., Panzeri M.M., Barbera M., Palumbo D., Sini C., Muffatti F., Andreasi V., Steidler S., Doglioni C. (2021). CT-derived radiomic features to discriminate histologic characteristics of pancreatic neuroendocrine tumors. Radiol. Med..

[41] Nardone V., Reginelli A., Grassi R., Boldrini L., Vacca G., D’Ippolito E., Annunziata S., Farchione A., Belfiore M.P., Desideri I. (2021). Delta radiomics: A systematic review. Radiol. Med..

[42] Cardobi N., Benetti G., Cardano G., Arena C., Micheletto C., Cavedon C., Montemezzi S. (2021). CT radiomic models to distinguish COVID-19 pneumonia from other interstitial pneumonias. Radiol. Med..

[43] Cellina M., Pirovano M., Ciocca M., Gibelli D., Floridi C., Oliva G. (2021). Radiomic analysis of the optic nerve at the first episode of acute optic neuritis: An indicator of optic nerve pathology and a predictor of visual recovery?. Radiol. Med..

[44] Liu J., Wang C., Guo W., Zeng P., Liu Y., Lang N., Yuan H. (2021). A preliminary study using spinal MRI-based radiomics to predict high-risk cytogenetic abnormalities in multiple myeloma. Radiol. Med..

[45] Paoletti M., Muzic S.I., Marchetti F., Farina L.M., Bastianello S., Pichiecchio A. (2021). Differential imaging of atypical demyelinating lesions of the central nervous system. Radiol. Med..

[46] Chianca V., Albano D., Messina C., Vincenzo G., Rizzo S., Del Grande F., Sconfienza L.M. (2021). An update in musculoskeletal tumors: From quantitative imaging to radiomics. Radiol. Med..

[47] Satake H., Ishigaki S., Ito R., Naganawa S. (2022). Radiomics in breast MRI: Current progress toward clinical application in the era of artificial intelligence. Radiol. Med..

[48] Qin H., Que Q., Lin P., Li X., Wang X.R., He Y., Chen J.Q., Yang H. (2021). Magnetic resonance imaging (MRI) radiomics of papillary thyroid cancer (PTC): A comparison of predictive performance of multiple classifiers modeling to identify cervical lymph node metastases before surgery. Radiol. Med..

[49] Karmazanovsky G., Gruzdev I., Tikhonova V., Kondratyev E., Revishvili A. (2021). Computed tomography-based radiomics approach in pancreatic tumors characterization. Radiol. Med..

[50] Gregucci F., Fiorentino A., Mazzola R., Ricchetti F., Bonaparte I., Surgo A., Figlia V., Carbonara R., Caliandro M., Ciliberti M.P. (2022). Radiomic analysis to predict local response in locally advanced pancreatic cancer treated with stereotactic body radiation therapy. Radiol. Med..

[51] Fusco R., Granata V., Maio F., Sansone M., Petrillo A. (2020). Textural radiomic features and time-intensity curve data analysis by dynamic contrast-enhanced MRI for early prediction of breast cancer therapy response: Preliminary data. Eur. Radiol. Exp..

[52] Van der Lubbe M.F.J.A., Vaidyanathan A., de Wit M., van den Burg E.L., Postma A.A., Bruintjes T.D., Bilderbeek-Beckers M.A.L., Dammeijer P.F.M., Bossche S.V., Van Rompaey V. (2022). A non-invasive, automated diagnosis of Menière’s disease using radiomics and machine learning on conventional magnetic resonance imaging: A multicentric, case-controlled feasibility study. Radiol. Med..

[53] Brunese L., Brunese M.C., Carbone M., Ciccone V., Mercaldo F., Santone A. (2022). Automatic PI-RADS assignment by means of formal methods. Radiol. Med..

[54] Vicini S., Bortolotto C., Rengo M., Ballerini D., Bellini D., Carbone I., Preda L., Laghi A., Coppola F., Faggioni L. (2022). A narrative review on current imaging applications of artificial intelligence and radiomics in oncology: Focus on the three most common cancers. Radiol. Med..

[55] Gao W., Wang W., Song D., Yang C., Zhu K., Zeng M., Rao S.X., Wang M. (2022). A predictive model integrating deep and radiomics features based on gadobenate dimeglumine-enhanced MRI for postoperative early recurrence of hepatocellular carcinoma. Radiol. Med..

[56] De Robertis R., Geraci L., Tomaiuolo L., Bortoli L., Beleù A., Malleo G., D’Onofrio M. (2022). Liver metastases in pancreatic ductal adenocarcinoma: A predictive model based on CT texture analysis. Radiol. Med..

[57] Yao F., Bian S., Zhu D., Yuan Y., Pan K., Pan Z., Feng X., Tang K., Yang Y. (2022). Machine learning-based radiomics for multiple primary prostate cancer biological characteristics prediction with 18F-PSMA-1007 PET: Comparison among different volume segmentation thresholds. Radiol. Med..

[58] Limkin E.J., Sun R., Dercle L., Zacharaki E.I., Robert C., Reuzé S., Schernberg A., Paragios N., Deutsch E., Ferté C. (2017). Promises and challenges for the implementation of computational medical imaging (radiomics) in oncology. Ann. Oncol..

[59] Cozzi D., Bicci E., Cavigli E., Danti G., Bettarini S., Tortoli P., Mazzoni L.N., Busoni S., Pradella S., Miele V. (2022). Radiomics in pulmonary neuroendocrine tumours (NETs). Radiol. Med..

[60] Danti G., Flammia F., Matteuzzi B., Cozzi D., Berti V., Grazzini G., Pradella S., Recchia L., Brunese L., Miele V. (2021). Gastrointestinal neuroendocrine neoplasms (GI-NENs): Hot topics in morphological, functional, and prognostic imaging. Radiol. Med..

[61] Faccioli N., Santi E., Foti G., D’Onofrio M. (2022). Cost-effectiveness analysis of including contrast-enhanced ultrasound in management of pancreatic cystic neoplasms. Radiol. Med..

[62] Brizi M.G., Perillo F., Cannone F., Tuzza L., Manfredi R. (2021). The role of imaging in acute pancreatitis. Radiol. Med..

[63] Guo C., Zhuge X., Wang Q., Xiao W., Wang Z., Wang Z., Feng Z., Chen X. (2018). The differentiation of pancreatic neuroendocrine carcinoma from pancreatic ductal adenocarcinoma: The values of CT imaging features and texture analysis. Cancer Imaging.

[64] Li J., Lu J., Liang P., Li A., Hu Y., Shen Y., Hu D., Li Z. (2018). Differentiation of atypical pancreatic neuroendocrine tumors from pancreatic ductal adenocarcinomas: Using whole-tumor CT texture analysis as quantitative biomarkers. Cancer Med..

[65] He M., Liu Z., Lin Y., Wan J., Li J., Xu K., Wang Y., Jin Z., Tian J., Xue H. (2019). Differentiation of atypical non-functional pancreatic neuroendocrine tumor and pancreatic ductal adenocarcinoma using CT based radiomics. Eur. J. Radiol..

[66] Shindo T., Fukukura Y., Umanodan T., Takumi K., Hakamada H., Nakajo M., Umanodan A., Ideue J., Kamimura K., Yoshiura T. (2016). Histogram Analysis of Apparent Diffusion Coefficient in Differentiating Pancreatic Adenocarcinoma and Neuroendocrine Tumor. Medicine.

[67] Han X., Yang J., Luo J., Chen P., Zhang Z., Alu A., Xiao Y., Ma X. (2021). Application of CT-Based Radiomics in Discriminating Pancreatic Cystadenomas from Pancreatic Neuroendocrine Tumors Using Machine Learning Methods. Front. Oncol..

[68] Ohara Y., Oda T., Hashimoto S., Akashi Y., Miyamoto R., Enomoto T., Satomi K., Morishita Y., Ohkohchi N. (2016). Pancreatic neuroendocrine tumor and solid-pseudopapillary neoplasm: Key immunohistochemical profiles for differential diagnosis. World J. Gastroenterol..

[69] Shi Y.J., Zhu H.T., Liu Y.L., Wei Y.Y., Qin X.B., Zhang X.Y., Li X.T., Sun Y.S. (2020). Radiomics Analysis Based on Diffusion Kurtosis Imaging and T2 Weighted Imaging for Differentiation of Pancreatic Neuroendocrine Tumors from Solid Pseudopapillary Tumors. Front. Oncol..

[70] Wang F.H., Zheng H.L., Li J.T., Li P., Zheng C.H., Chen Q.Y., Huang C.M., Xie J.W. (2022). Prediction of recurrence-free survival and adjuvant therapy benefit in patients with gastrointestinal stromal tumors based on radiomics features. Radiol. Med..

[71] Palatresi D., Fedeli F., Danti G., Pasqualini E., Castiglione F., Messerini L., Massi D., Bettarini S., Tortoli P., Busoni S. (2022). Correlation of CT radiomic features for GISTs with pathological classification and molecular subtypes: Preliminary and monocentric experience. Radiol. Med..

[72] Demirjian N.L., Varghese B.A., Cen S.Y., Hwang D.H., Aron M., Siddiqui I., Fields B.K.K., Lei X., Yap F.Y., Rivas M. (2022). CT-based radiomics stratification of tumor grade and TNM stage of clear cell renal cell carcinoma. Eur. Radiol..

[73] Xue K., Liu L., Liu Y., Guo Y., Zhu Y., Zhang M. (2022). Radiomics model based on multi-sequence MR images for predicting preoperative immunoscore in rectal cancer. Radiol. Med..

[74] Chiloiro G., Cusumano D., de Franco P., Lenkowicz J., Boldrini L., Carano D., Barbaro B., Corvari B., Dinapoli N., Giraffa M. (2022). Does restaging MRI radiomics analysis improve pathological complete response prediction in rectal cancer patients? A prognostic model development. Radiol. Med..

[75] Cusumano D., Meijer G., Lenkowicz J., Chiloiro G., Boldrini L., Masciocchi C., Dinapoli N., Gatta R., Casà C., Damiani A. (2021). A field strength independent MR radiomics model to predict pathological complete response in locally advanced rectal cancer. Radiol. Med..

[76] Bracci S., Dolciami M., Trobiani C., Izzo A., Pernazza A., D’Amati G., Manganaro L., Ricci P. (2021). Quantitative CT texture analysis in predicting PD-L1 expression in locally advanced or metastatic NSCLC patients. Radiol. Med..

[77] Tomori Y., Yamashiro T., Tomita H., Tsubakimoto M., Ishigami K., Atsumi E., Murayama S. (2020). CT radiomics analysis of lung cancers: Differentiation of squamous cell carcinoma from adenocarcinoma, a correlative study with FDG uptake. Eur. J. Radiol..

[78] Autorino R., Gui B., Panza G., Boldrini L., Cusumano D., Russo L., Nardangeli A., Persiani S., Campitelli M., Ferrandina G. (2022). Radiomics-based prediction of two-year clinical outcome in locally advanced cervical cancer patients undergoing neoadjuvant chemoradiotherapy. Radiol. Med..

[79] Gitto S., Bologna M., Corino V.D.A., Emili I., Albano D., Messina C., Armiraglio E., Parafioriti A., Luzzati A., Mainardi L. (2022). Diffusion-weighted MRI radiomics of spine bone tumors: Feature stability and machine learning-based classification performance. Radiol. Med..

[80] Han D., Chen Y., Li X., Li W., Zhang X., He T., Yu Y., Dou Y., Duan H., Yu N. (2023). Development and validation of a 3D-convolutional neural network model based on chest CT for differentiating active pulmonary tuberculosis from community–acquired pneumonia. Radiol. Med..

[81] Zerunian M., Pucciarelli F., Caruso D., Polici M., Masci B., Guido G., De Santis D., Polverari D., Principessa D., Benvenga A. (2022). Artificial intelligence based image quality enhancement in liver MRI: A quantitative and qualitative evaluation. Radiol. Med..

[82] Cipollari S., Pecoraro M., Forookhi A., Laschena L., Bicchetti M., Messina E., Lucciola S., Catalano C., Panebianco V. (2022). Biparametric prostate MRI: Impact of a deep learning-based software and of quantitative ADC values on the inter-reader agreement of experienced and inexperienced readers. Radiol. Med..

[83] Ventura C., Baldassarre S., Cerimele F., Pepi L., Marconi E., Ercolani P., Floridi C., Argalia G., Goteri G., Giovagnoni A. (2022). 2D shear wave elastography in evaluation of prognostic factors in breast cancer. Radiol. Med..

[84] Mega S., Fiore M., Carpenito M., Novembre M.L., Miele M., Trodella L.E., Grigioni F., Ippolito E., Ramella S. (2022). Early GLS changes detection after chemoradiation in locally advanced non-small cell lung cancer (NSCLC). Radiol. Med..

[85] Lo Casto A., Cannella R., Taravella R., Cordova A., Matta D., Campisi G., Attanasio M., Rinaldi G., Rodolico V. (2022). Diagnostic and prognostic value of magnetic resonance imaging in the detection of tumor depth of invasion and bone invasion in patients with oral cavity cancer. Radiol. Med..

[86] Fan Y., Zhao Z., Wang X., Ai H., Yang C., Luo Y., Jiang X. (2022). Radiomics for prediction of response to EGFR-TKI based on metastasis/brain parenchyma (M/BP)-interface. Radiol. Med..

[87] Bartolotta T.V., Orlando A.A.M., Dimarco M., Zarcaro C., Ferraro F., Cirino A., Matranga D., Vieni S., Cabibi D. (2022). Diagnostic performance of 2D-shear wave elastography in the diagnosis of breast cancer: A clinical appraisal of cutoff values. Radiol. Med..

[88] Sun J., Li H., Gao J., Li J., Li M., Zhou Z., Peng Y. (2021). Performance evaluation of a deep learning image reconstruction (DLIR) algorithm in “double low” chest CTA in children: A feasibility study. Radiol. Med..

[89] Chiti G., Grazzini G., Flammia F., Matteuzzi B., Tortoli P., Bettarini S., Pasqualini E., Granata V., Busoni S., Messserini L. (2022). Gastroenteropancreatic neuroendocrine neoplasms (GEP-NENs): A radiomic model to predict tumor grade. Radiol. Med..

[90] Luo Y., Chen X., Chen J., Song C., Shen J., Xiao H., Chen M., Li Z.P., Huang B., Feng S.T. (2020). Preoperative Prediction of Pancreatic Neuroendocrine Neoplasms Grading Based on Enhanced Computed Tomography Imaging: Validation of Deep Learning with a Convolutional Neural Network. Neuroendocrinology.

[91] Pavel M., Öberg K., Falconi M., Krenning E.P., Sundin A., Perren A., Berruti A., ESMO Guidelines Committee (2020). Gastroenteropancreatic neuroendocrine neoplasms: ESMO Clinical Practice Guidelines for diagnosis, treatment and follow-up. Ann. Oncol..

[92] Song C., Wang M., Luo Y., Chen J., Peng Z., Wang Y., Zhang H., Li Z.P., Shen J., Huang B. (2021). Predicting the recurrence risk of pancreatic neuroendocrine neoplasms after radical resection using deep learning radiomics with preoperative computed tomography images. Ann. Transl. Med..

[93] Pereira J.A., Rosado E., Bali M., Metens T., Chao S.L. (2015). Pancreatic neuroendocrine tumors: Correlation between histogram analysis of apparent diffusion coefficient maps and tumor grade. Abdom. Imaging.

[94] Granata V., Grassi R., Fusco R., Setola S.V., Palaia R., Belli A., Miele V., Brunese L., Grassi R., Petrillo A. (2020). Assessment of Ablation Therapy in Pancreatic Cancer: The Radiologist’s Challenge. Front. Oncol..

[95] Cholangiocarcinoma Working Group (2020). Italian Clinical Practice Guidelines on Cholangiocarcinoma—Part I: Classification, diagnosis and staging. Dig. Liver Dis..

[96] Guo C., Zhuge X., Wang Z., Wang Q., Sun K., Feng Z., Chen X. (2019). Textural analysis on contrast-enhanced CT in pancreatic neuroendocrine neoplasms: Association with WHO grade. Abdom. Radiol..

[97] Canellas R., Burk K.S., Parakh A., Sahani D.V. (2018). Prediction of Pancreatic Neuroendocrine Tumor Grade Based on CT Features and Texture Analysis. AJR Am. J. Roentgenol..

[98] De Robertis R., Maris B., Cardobi N., Tinazzi Martini P., Gobbo S., Capelli P., Ortolani S., Cingarlini S., Paiella S., Landoni L. (2018). Can histogram analysis of MR images predict aggressiveness in pancreatic neuroendocrine tumors?. Eur. Radiol..

[99] Calandrelli R., Boldrini L., Tran H.E., Quinci V., Massimi L., Pilato F., Lenkowicz J., Votta C., Colosimo C. (2022). CT-based radiomics modeling for skull dysmorphology severity and surgical outcome prediction in children with isolated sagittal synostosis: A hypothesis-generating study. Radiol. Med..

[100] Granata V., Fusco R., Risi C., Ottaiano A., Avallone A., De Stefano A., Grimm R., Grassi R., Brunese L., Izzo F. (2020). Diffusion-Weighted MRI and Diffusion Kurtosis Imaging to Detect RAS Mutation in Colorectal Liver Metastasis. Cancers.

[101] Granata V., Fusco R., Avallone A., Catalano O., Filice F., Leongito M., Palaia R., Izzo F., Petrillo A. (2017). Major and ancillary magnetic resonance features of LI-RADS to assess HCC: An overview and update. Infect. Agents Cancer.

[102] Granata V., Fusco R., Sansone M., Grassi R., Maio F., Palaia R., Tatangelo F., Botti G., Grimm R., Curley S. (2020). Magnetic resonance imaging in the assessment of pancreatic cancer with quantitative parameter extraction by means of dynamic contrast-enhanced magnetic resonance imaging, diffusion kurtosis imaging and intravoxel incoherent motion diffusion-weighted imaging. Therap. Adv. Gastroenterol..

[103] Granata V., Fusco R., Setola S.V., Picone C., Vallone P., Belli A., Incollingo P., Albino V., Tatangelo F., Izzo F. (2019). Microvascular invasion and grading in hepatocellular carcinoma: Correlation with major and ancillary features according to LIRADS. Abdom. Radiol..

[104] Fusco R., Sansone M., Granata V., Grimm R., Pace U., Delrio P., Tatangelo F., Botti G., Avallone A., Pecori B. (2019). Diffusion and perfusion MR parameters to assess preoperative short-course radiotherapy response in locally advanced rectal cancer: A comparative explorative study among Standardized Index of Shape by DCE-MRI, intravoxel incoherent motion- and diffusion kurtosis imaging-derived parameters. Abdom. Radiol..

[105] Rea G., De Martino M., Capaccio A., Dolce P., Valente T., Castaldo S., Canora A., Lassandro F., Bocchino M. (2021). Comparative analysis of density histograms and visual scores in incremental and volumetric high-resolution computed tomography of the chest in idiopathic pulmonary fibrosis patients. Radiol. Med..

[106] Zhang T., Zhang Y., Liu X., Xu H., Chen C., Zhou X., Liu Y., Ma X. (2021). Application of Radiomics Analysis Based on CT Combined with Machine Learning in Diagnostic of Pancreatic Neuroendocrine Tumors Patient’s Pathological Grades. Front. Oncol..

[107] Reinert C.P., Baumgartner K., Hepp T., Bitzer M., Horger M. (2020). Complementary role of computed tomography texture analysis for differentiation of pancreatic ductal adenocarcinoma from pancreatic neuroendocrine tumors in the portal-venous enhancement phase. Abdom. Radiol..

[108] Bian Y., Jiang H., Ma C., Wang L., Zheng J., Jin G., Lu J. (2020). CT-Based Radiomics Score for Distinguishing Between Grade 1 and Grade 2 Nonfunctioning Pancreatic Neuroendocrine Tumors. AJR Am. J. Roentgenol..

[109] Choi T.W., Kim J.H., Yu M.H., Park S.J., Han J.K. (2018). Pancreatic neuroendocrine tumor: Prediction of the tumor grade using CT findings and computerized texture analysis. Acta Radiol..

[110] Gu D., Hu Y., Ding H., Wei J., Chen K., Liu H., Zeng M., Tian J. (2019). CT radiomics may predict the grade of pancreatic neuroendocrine tumors: A multicenter study. Eur. Radiol..

[111] Liang W., Yang P., Huang R., Xu L., Wang J., Liu W., Zhang L., Wan D., Huang Q., Lu Y. (2019). A Combined Nomogram Model to Preoperatively Predict Histologic Grade in Pancreatic Neuroendocrine Tumors. Clin. Cancer Res..

[112] Fusco R., Granata V., Grazzini G., Pradella S., Borgheresi A., Bruno A., Palumbo P., Bruno F., Grassi R., Giovagnoni A. (2022). Radiomics in medical imaging: Pitfalls and challenges in clinical management. Jpn. J. Radiol..

[113] Scapicchio C., Gabelloni M., Barucci A., Cioni D., Saba L., Neri E. (2021). A deep look into radiomics. Radiol. Med..

[114] Lin J.X., Wang F.H., Wang Z.K., Wang J.B., Zheng C.H., Li P., Huang C.M., Xie J.W. (2023). Prediction of the mitotic index and preoperative risk stratification of gastrointestinal stromal tumors with CT radiomic features. Radiol. Med..

[115] Granata V., Grassi R., Fusco R., Galdiero R., Setola S.V., Palaia R., Belli A., Silvestro L., Cozzi D., Brunese L. (2021). Pancreatic cancer detection and characterization: State of the art and radiomics. Eur. Rev. Med. Pharmacol. Sci..

[116] Chen L., Wang W., Jin K., Yuan B., Tan H., Sun J., Guo Y., Luo Y., Feng S.T., Yu X. (2023). Special issue “The advance of solid tumor research in China”: Prediction of Sunitinib efficacy using computed tomography in patients with pancreatic neuroendocrine tumors. Int. J. Cancer.

[117] Song Y., Gao S., Tan W., Qiu Z., Zhou H., Zhao Y. (2018). Multiple Machine Learnings Revealed Similar Predictive Accuracy for Prognosis of PNETs from the Surveillance, Epidemiology, and End Result Database. J. Cancer..

[118] Werner R.A., Lapa C., Ilhan H., Higuchi T., Buck A.K., Lehner S., Bartenstein P., Bengel F., Schatka I., Muegge D.O. (2017). Survival prediction in patients undergoing radionuclide therapy based on intratumoral somatostatin-receptor heterogeneity. Oncotarget.

[119] Martini I., Polici M., Zerunian M., Panzuto F., Rinzivillo M., Landolfi F., Magi L., Caruso D., Eid M., Annibale B. (2020). CT texture analysis of liver metastases in PNETs versus NPNETs: Correlation with histopathological findings. Eur. J. Radiol..

[120] Önner H., Abdülrezzak Ü., Tutuş A. (2020). Could the skewness and kurtosis texture parameters of lesions obtained from pretreatment Ga-68 DOTA-TATE PET/CT images predict receptor radionuclide therapy response in patients with gastroenteropancreatic neuroendocrine tumors?. Nucl. Med. Commun..

[121] An P., Zhang J., Li M., Duan P., He Z., Wang Z., Feng G., Guo H., Li X., Qin P. (2022). Clinical Data-CT Radiomics-Based Model for Predicting Prognosis of Patients with Gastrointestinal Pancreatic Neuroendocrine Neoplasms (GP-NENs). Comput. Math. Methods Med..

[122] Preuss K., Thach N., Liang X., Baine M., Chen J., Zhang C., Du H., Yu H., Lin C., Hollingsworth M.A. (2022). Using Quantitative Imaging for Personalized Medicine in Pancreatic Cancer: A Review of Radiomics and Deep Learning Applications. Cancers.

[123] Vesterinen T., Säilä J., Blom S., Pennanen M., Leijon H., Arola J. (2022). Automated assessment of Ki-67 proliferation index in neuroendocrine tumors by deep learning. APMIS.

[124] Govind D., Jen K.Y., Matsukuma K., Gao G., Olson K.A., Gui D., Wilding G.E., Border S.P., Sarder P. (2020). Improving the accuracy of gastrointestinal neuroendocrine tumor grading with deep learning. Sci. Rep..

[125] Granata V., Fusco R., Setola S.V., Galdiero R., Maggialetti N., Patrone R., Ottaiano A., Nasti G., Silvestro L., Cassata A. (2023). Correction: Colorectal liver metastases patients prognostic assessment: Prospects and limits of radiomics and radiogenomics. Infect. Agents Cancer.

[126] Salvestrini V., Becherini C., Desideri I., Caprara L., Mariotti M., Banini M., Pierossi N., Scotti V., Livi L., Bonomo P. (2022). The impact of patient preference in the treatment algorithm for recurrent/metastatic head and neck squamous cell carcinoma. Radiol. Med..

[127] Granata V., Fusco R., De Muzio F., Cutolo C., Setola S.V., Dell’Aversana F., Ottaiano A., Nasti G., Grassi R., Pilone V. (2022). EOB-MR Based Radiomics Analysis to Assess Clinical Outcomes following Liver Resection in Colorectal Liver Metastases. Cancers.

[128] Francolini G., Jereczek-Fossa B.A., Di Cataldo V., Simontacchi G., Marvaso G., Gandini S., Corso F., Ciccone L.P., Zerella M.A., Gentile P. (2022). Stereotactic or conventional radiotherapy for macroscopic prostate bed recurrence: A propensity score analysis. Radiol. Med..

[129] Granata V., Fusco R., De Muzio F., Cutolo C., Setola S.V., Dell’Aversana F., Grassi F., Belli A., Silvestro L., Ottaiano A. (2022). Radiomics and machine learning analysis based on magnetic resonance imaging in the assessment of liver mucinous colorectal metastases. Radiol. Med..

[130] Orlhac F., Nioche C., Klyuzhin I., Rahmim A., Buvat I. (2021). Radiomics in PET Imaging: A Practical Guide for Newcomers. PET Clin..

[131] Avanzo M., Stancanello J., El Naqa I. (2017). Beyond imaging: The promise of radiomics. Phys. Med..

[132] Da-Ano R., Visvikis D., Hatt M. (2020). Harmonization strategies for multicenter radiomics investigations. Phys. Med. Biol..

[133] Bogowicz M., Vuong D., Huellner M.W., Pavic M., Andratschke N., Gabrys H.S., Guckenberger M., Tanadini-Lang S. (2019). CT radiomics and PET radiomics: Ready for clinical implementation?. Q. J. Nucl. Med. Mol. Imaging.

[134] Arimura H., Soufi M., Kamezawa H., Ninomiya K., Yamada M. (2019). Radiomics with artificial intelligence for precision medicine in radiation therapy. J. Radiat. Res..

[135] Neri E., Del Re M., Paiar F., Erba P., Cocuzza P., Regge D., Danesi R. (2018). Radiomics and liquid biopsy in oncology: The holons of systems medicine. Insights Imaging.

[136] Ji G.W., Wang K., Xia Y.X., Li X.C., Wang X.H. (2020). Application and challenge of radiomics technique in the era of precision medicine for hepatobiliary disease. Zhonghua Wai Ke Za Zhi.

[137] Yip S.S., Aerts H.J. (2016). Applications and limitations of radiomics. Phys. Med. Biol..

[138] Rizzo S., Botta F., Raimondi S., Origgi D., Fanciullo C., Morganti A.G., Bellomi M. (2018). Radiomics: The facts and the challenges of image analysis. Eur. Radiol. Exp..

[139] Masci G.M., Iafrate F., Ciccarelli F., Pambianchi G., Panebianco V., Pasculli P., Ciardi M.R., Mastroianni C.M., Ricci P., Catalano C. (2021). Tocilizumab effects in COVID-19 pneumonia: Role of CT texture analysis in quantitative assessment of response to therapy. Radiol. Med..

[140] Ligero M., Jordi-Ollero O., Bernatowicz K., Garcia-Ruiz A., Delgado-Muñoz E., Leiva D., Mast R., Suarez C., Sala-Llonch R., Calvo N. (2021). Minimizing acquisition-related radiomics variability by image resampling and batch effect correction to allow for large-scale data analysis. Eur. Radiol..

[141] Spinelli M.S., Balbaa M.F., Gallazzi M.B., Eid M.E., Kotb H.T., Shafei M.E., Ierardi A.M., Daolio P.A., Barile A., Carrafiello G. (2022). Role of percutaneous CT-guided radiofrequency ablation in treatment of intra-articular, in close contact with cartilage and extra-articular osteoid osteomas: Comparative analysis and new classification system. Radiol. Med..

[142] Caruso D., Polici M., Rinzivillo M., Zerunian M., Nacci I., Marasco M., Magi L., Tarallo M., Gargiulo S., Iannicelli E. (2022). CT-based radiomics for prediction of therapeutic response to Everolimus in metastatic neuroendocrine tumors. Radiol. Med..

[143] Han D., Yu N., Yu Y., He T., Duan X. (2022). Performance of CT radiomics in predicting the overall survival of patients with stage III clear cell renal carcinoma after radical nephrectomy. Radiol. Med..

[144] Donati O.F., Hany T.F., Reiner C.S., von Schulthess G.K., Marincek B., Seifert B., Weishaupt D. (2010). Value of retrospective fusion of PET and MR images in detection of hepatic metastases: Comparison with 18F-FDG PET/CT and Gd-EOB-DTPA-enhanced MRI. J. Nucl. Med..

[145] Masci G.M., Ciccarelli F., Mattei F.I., Grasso D., Accarpio F., Catalano C., Laghi A., Sammartino P., Iafrate F. (2022). Role of CT texture analysis for predicting peritoneal metastases in patients with gastric cancer. Radiol. Med..

[146] Scheckenbach K. (2018). Radiomics: Big Data Instead of Biopsies in the Future?. Laryngorhinootologie.

[147] Zanfardino M., Franzese M., Pane K., Cavaliere C., Monti S., Esposito G., Salvatore M., Aiello M. (2019). Bringing radiomics into a multi-omics framework for a comprehensive genotype-phenotype characterization of oncological diseases. J. Transl. Med..

[148] Grassi R., Belfiore M.P., Montanelli A., Patelli G., Urraro F., Giacobbe G., Fusco R., Granata V., Petrillo A., Sacco P. (2021). COVID-19 pneumonia: Computer-aided quantification of healthy lung parenchyma, emphysema, ground glass and consolidation on chest computed tomography (CT). Radiol. Med..

[149] Lafata K.J., Wang Y., Konkel B., Yin F.F., Bashir M.R. (2022). Radiomics: A primer on high-throughput image phenotyping. Abdom. Radiol..

[150] Lenga L., Bernatz S., Martin S.S., Booz C., Solbach C., Mulert-Ernst R., Vogl T.J., Leithner D. (2021). Iodine Map Radiomics in Breast Cancer: Prediction of Metastatic Status. Cancers.

[151] Sansone M., Marrone S., Di Salvio G., Belfiore M.P., Gatta G., Fusco R., Vanore L., Zuiani C., Grassi F., Vietri M.T. (2022). Comparison between two packages for pectoral muscle removal on mammographic images. Radiol. Med..

[152] Granata V., Faggioni L., Grassi R., Fusco R., Reginelli A., Rega D., Maggialetti N., Buccicardi D., Frittoli B., Rengo M. (2022). Structured reporting of computed tomography in the staging of colon cancer: A Delphi consensus proposal. Radiol. Med..

[153] Borghesi A., Sverzellati N., Polverosi R., Balbi M., Baratella E., Busso M., Calandriello L., Cortese G., Farchione A., Iezzi R. (2021). Impact of the COVID-19 pandemic on the selection of chest imaging modalities and reporting systems: A survey of Italian radiologists. Radiol. Med..

[154] Pizzini F.B., Conti E., Bianchetti A., Splendiani A., Fusco D., Caranci F., Bozzao A., Landi F., Gandolfo N., Farina L. (2022). Radiological assessment of dementia: The Italian inter-society consensus for a practical and clinically oriented guide to image acquisition, evaluation, and reporting. Radiol. Med..

[155] Rengo M., Boru C.E., Badia S., Iossa A., Bellini D., Picchia S., Panvini N., Carbone I., Silecchia G., Laghi A. (2021). Preoperative measurement of the hiatal surface with MDCT: Impact on surgical planning. Radiol. Med..

[156] Neri E., Granata V., Montemezzi S., Belli P., Bernardi D., Brancato B., Caumo F., Calabrese M., Coppola F., Cossu E. (2022). Structured reporting of x-ray mammography in the first diagnosis of breast cancer: A Delphi consensus proposal. Radiol. Med..

[157] Frix A.N., Cousin F., Refaee T., Bottari F., Vaidyanathan A., Desir C., Vos W., Walsh S., Occhipinti M., Lovinfosse P. (2021). Radiomics in Lung Diseases Imaging: State-of-the-Art for Clinicians. J. Pers. Med..

[158] Granata V., Fusco R., Catalano O., Avallone A., Palaia R., Botti G., Tatangelo F., Granata F., Cascella M., Izzo F. (2017). Diagnostic accuracy of magnetic resonance, computed tomography and contrast enhanced ultrasound in radiological multimodality assessment of peribiliary liver metastases. PLoS ONE.

